# Malignant Melanocytic Matricoma: A Rare Skin Tumor That Can Clinically Mimic Melanoma

**DOI:** 10.7759/cureus.34105

**Published:** 2023-01-23

**Authors:** Layla Tahiri Elousrouti, Imane Fadlallah, Meryem Soughi, Houda Elabbad, Hanane Baybay, Fatima Zahra Mernissi, Hinde Elfatemi, Laila Chbani, Nawal Hammas

**Affiliations:** 1 Department of Pathology, Hassan II University Hospital, Sidi Mohamed Ben Abdellah University, Biomedical and Translational Research Laboratory, Faculty of Medicine and Pharmacy, Fez, MAR; 2 Department of Pathology, Hassan II University Hospital, Sidi Mohamed Ben Abdellah University, Faculty of Medicine and Pharmacy, Fez, MAR; 3 Department of Dermatology, Hassan II University Hospital, Sidi Mohamed Ben Abdellah University, Faculty of Medicine and Pharmacy, Fez, MAR

**Keywords:** beta-catenin antibody, dendritic melanocyte, malignant matricoma, carcinoma, matrical differentiation, skin appendage tumor

## Abstract

Malignant melanocytic matricoma (MMM) is an extremely rare skin malignant neoplasm composed of epithelial cells with matrical differentiation and dendritic melanocytes. We found only 11 cases reported in the literature to date according to the databases consulted (PubMed/Medline, Scopus, and Web of Science). Here, we report a case of MMM in an 86-year-old woman. A histological examination showed a dermal tumor with a deep infiltrative pattern, without an epidermal connection. On immunohistochemical staining, tumor cells were positive for cytokeratin AE1/AE3, p63, and beta-catenin (nuclear and cytoplasmic staining) and negative for HMB45, Melan-A, S-100 protein, and androgen receptor. Melanic antibodies highlighted scattered dendritic melanocytes in tumor sheets. The findings did not support the diagnosis of melanoma, poorly differentiated sebaceous carcinoma, and basal cell carcinoma, but supported the diagnosis of MMM.

## Introduction

Malignant melanocytic matricoma (MMM) is considered the malignant counterpart of melanotic matricoma which is a rare benign skin adnexal neoplasm composed of epithelial cells exhibiting matrical differentiation admixed with pigmented dendritic melanocytes [1]. MMM is even rarer than benign melanotic matricoma [1]. Although features for differentiating benign and MMM are controversial, we found marked cytological atypia, infiltrative growth pattern, necrosis, ulceration, recurrence, and metastasis. However, a high mitotic count would not constitute a criterion of aggressive behavior [2]. This tumor predominates in the head and neck, is more often reported in the elderly, and presents as a solitary pigmented nodule occurring on sun-damaged skin, which leads to a clinical diagnosis of melanoma or pigmented basal cell carcinoma. The histological analysis shows a dermal proliferation of atypical matrix epithelial cells admixed with varying proportions of pigmented dendritic melanocytes [3]. Here, we report the 12th reported case of MMM in an 86-year-old woman with sun-damaged skin who presented with a pigmented nodular tumor that mistakenly suggested melanoma, pigmented basal cell carcinoma, or trichoblastoma.

## Case presentation

**Figure 1:**
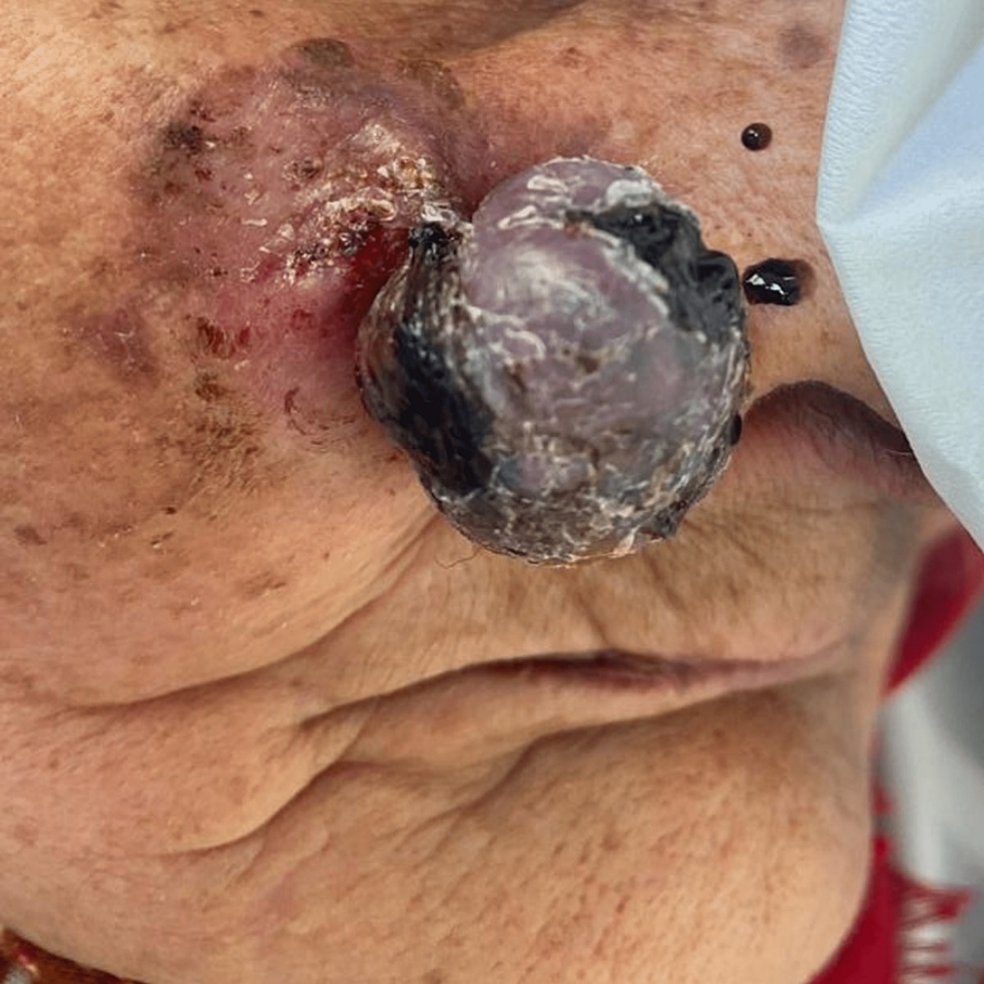
The pigmented nodular and blackish tumor with a pedunculated and infiltrated base measuring 4 cm.

**Figure 2:**
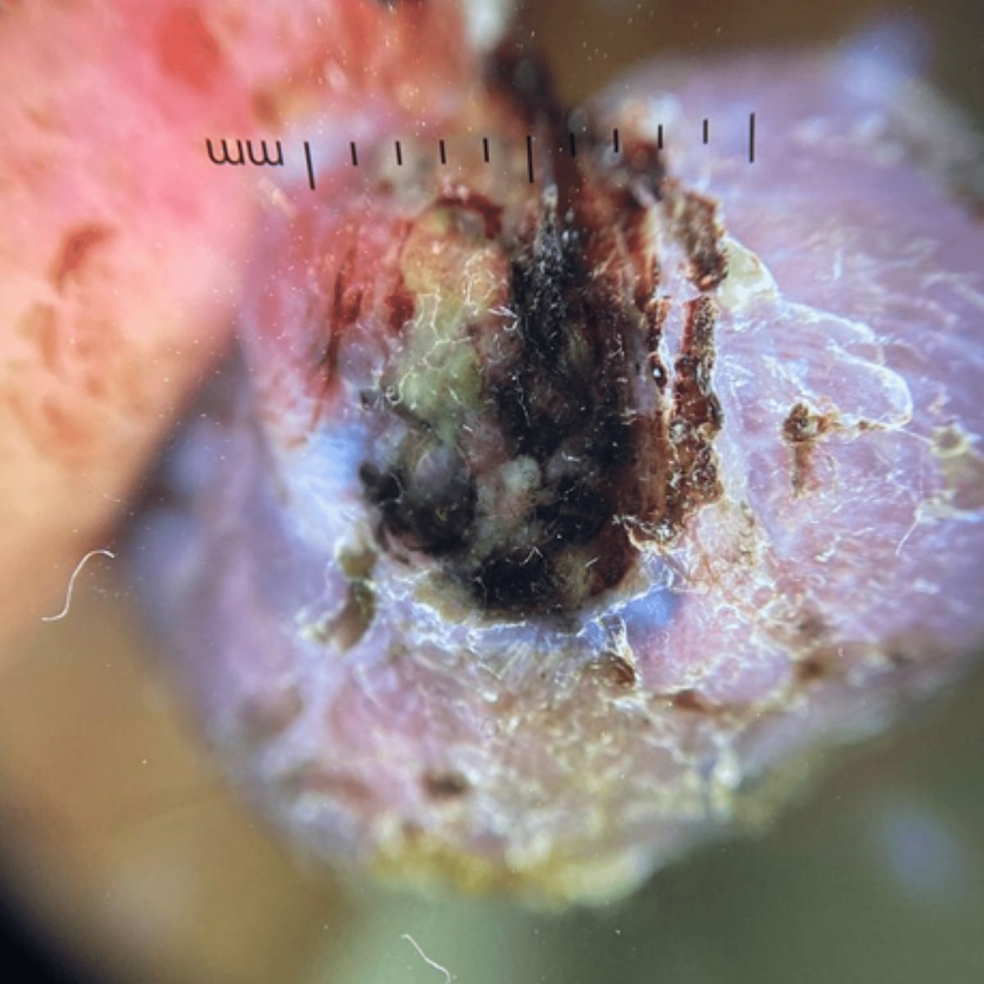
Dermoscopy reveals a chaotic pattern with a blue-white veil and tree-trunk vascular pattern.

An 86-year-old woman with the risk factor of chronic sun exposure consulted for a pigmented nodular tumor surmounted by scales on the right cheek. It was progressively increasing in size for three years and bled on contact for a few months. On clinical examination, we found a nodular and blackish tumor of firm consistency with a pedunculated and infiltrated base measuring 4 cm (Figure 1). Dermoscopy revealed a chaotic pattern with a blue-white veil and tree-trunk vascular pattern (Figure 2).

**Figure 3:**
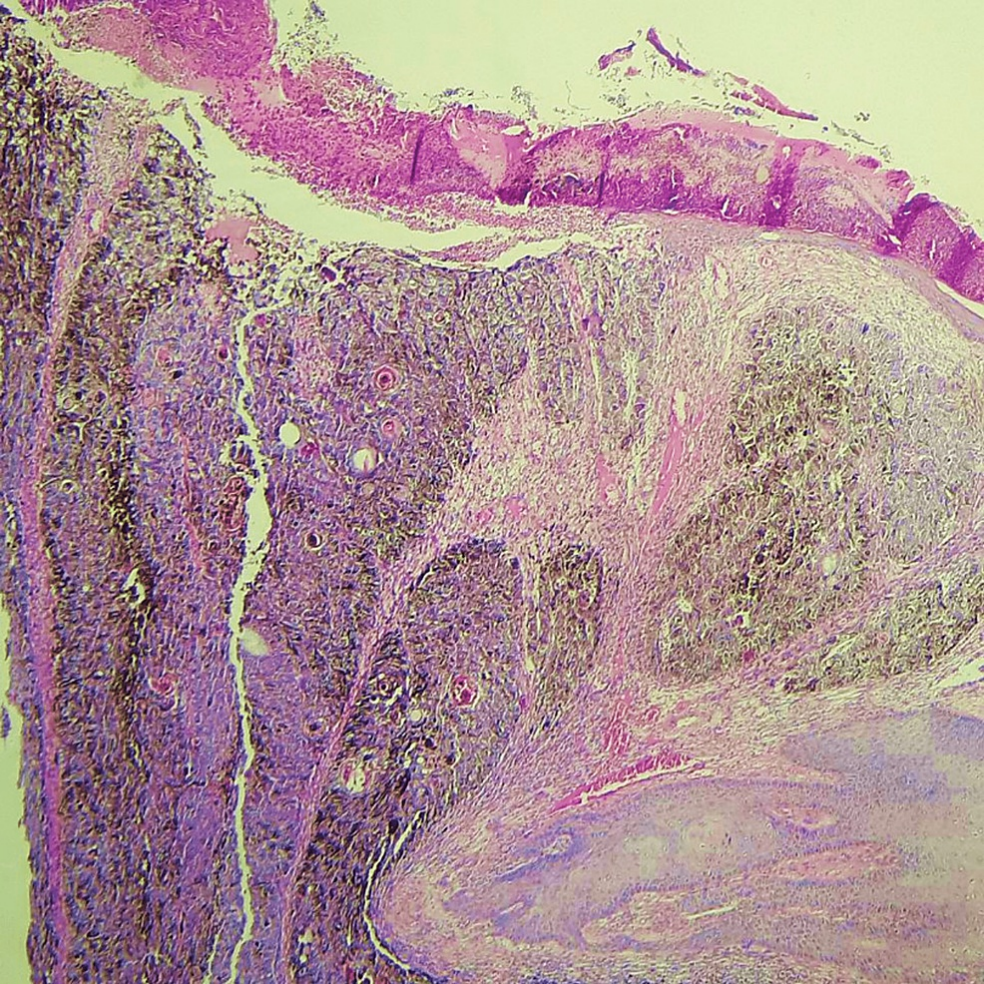
Histological examination shows an ulcerated skin lesion whose dermis contains a biphasic, basaloid, and pigmented tumor (hematoxylin-eosin-saffron ×100).

**Figure 4:**
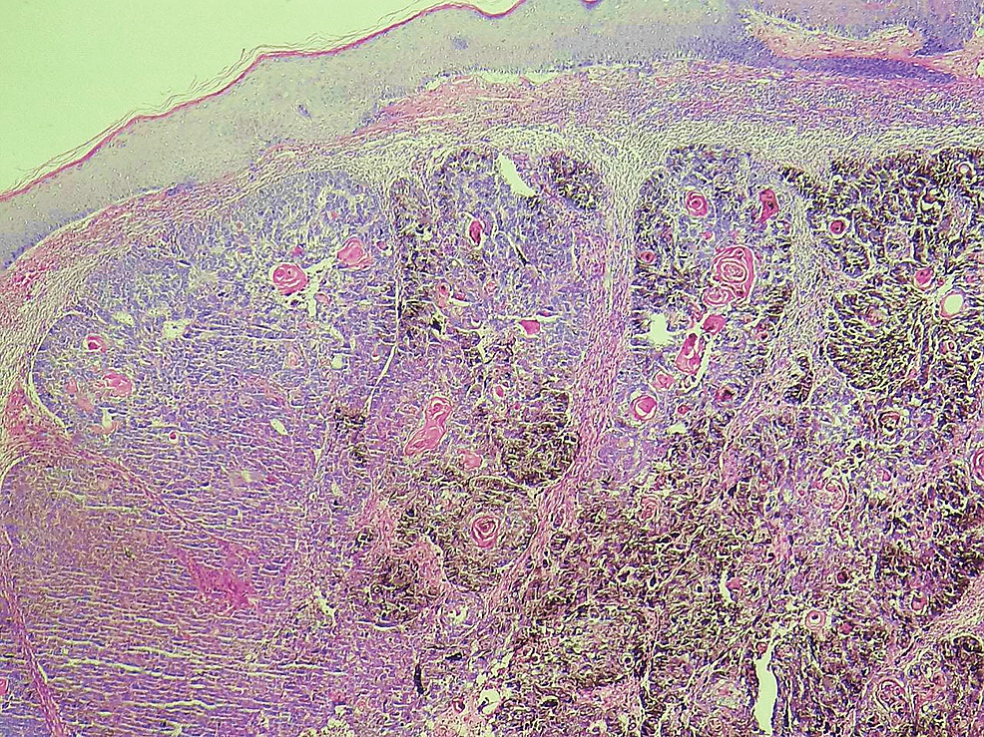
Histological examination shows adermal biphasic, basaloid, and pigmented tumor arranged in lobules and sheets without connection with the epidermis (hematoxylin-eosin-saffron ×100).

Suggested clinical diagnoses included melanoma, pigmented basal cell carcinoma, or trichoblastoma. Excision of the pedunculated nodule with its base was performed. The sample was fixed in 10% tamponed formalin, impregnated, and then included in a paraffin block. Subsequently, 4 μm sections were made and stained with standard hematoxylin-eosin-saffron (HES) staining. Optical microscopic examination showed an ulcerated skin lesion whose dermis contained a pigmented tumor arranged in lobules and sheets without connection with the epidermis (Figures 3, 4).

**Figure 5:**
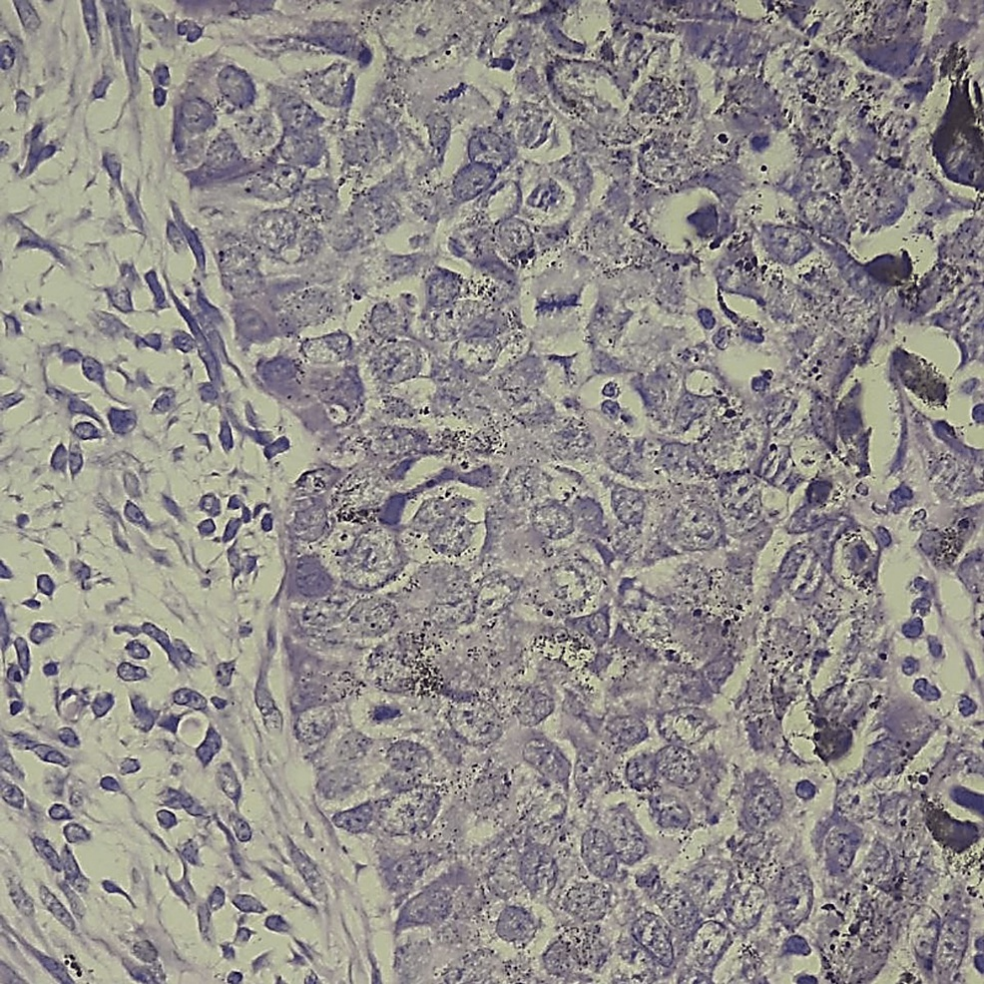
Epithelial tumor cells are basaloid, with a pleomorphic nucleus, prominent nucleolus, and basophilic-reduced cytoplasm. Atypical mitotic figures are noted (hematoxylin-eosin-saffron ×400).

**Figure 6:**
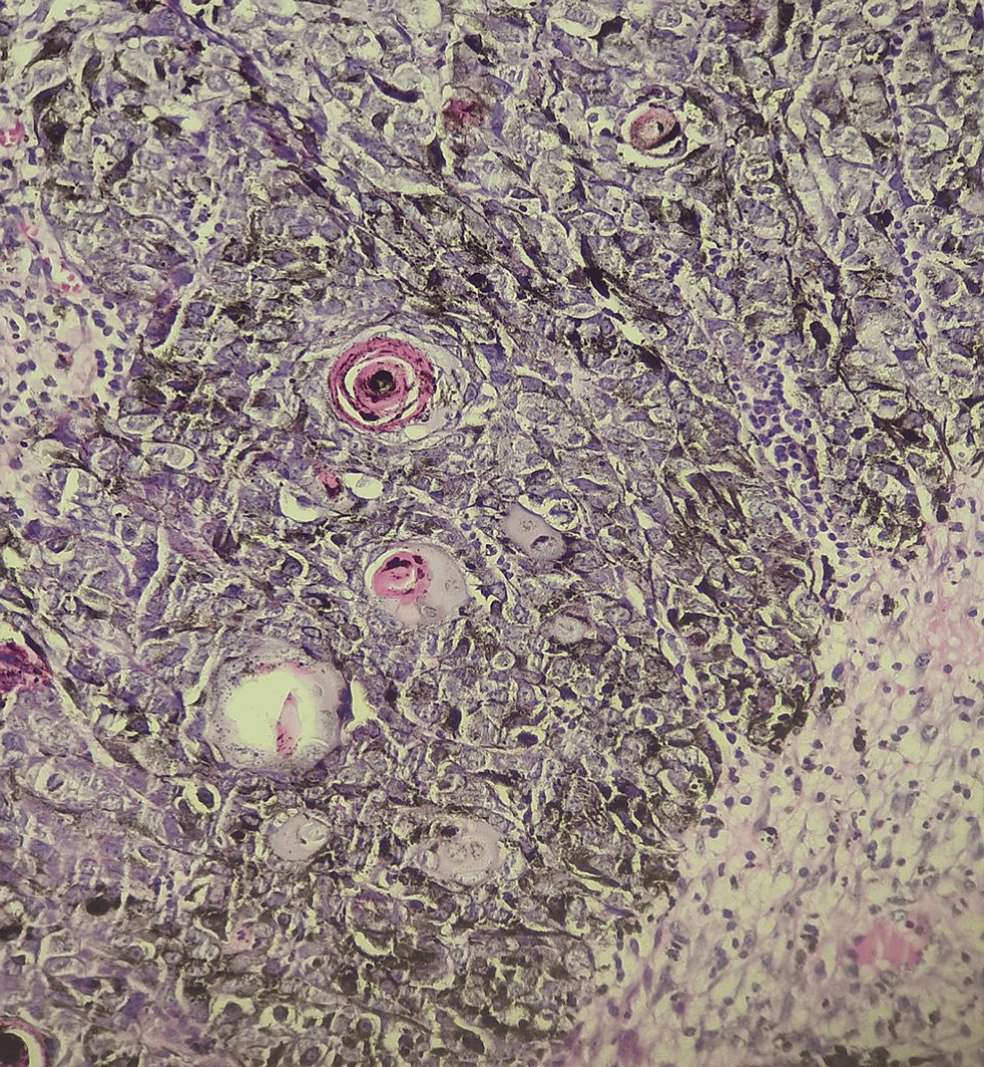
Epithelial basaloid cells are mixed with numerous heavily pigmented dendritic melanocytes (hematoxylin-eosin-saffron ×200).

**Figure 7:**
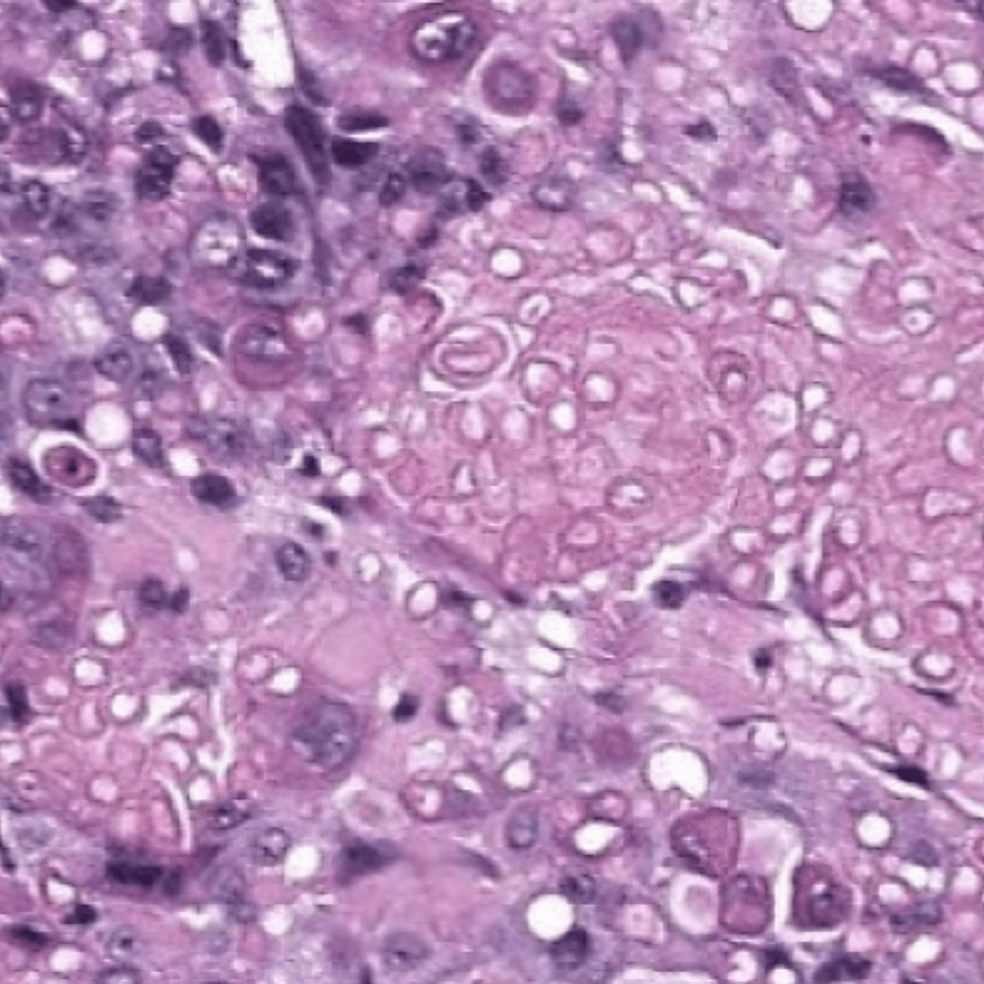
Clusters of ghost cells are noted (hematoxylin-eosin-saffron ×400).

**Figure 8:**
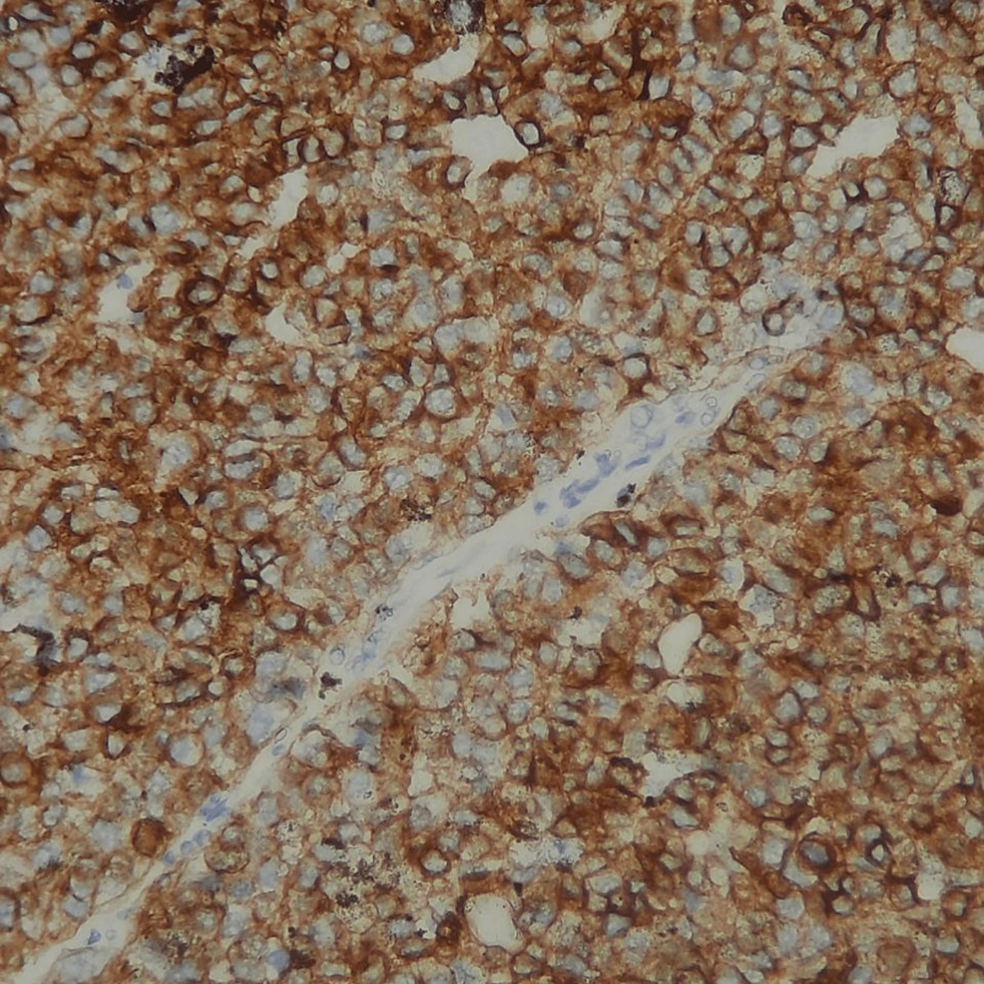
Cytoplasmic positive immunostaining by cytokeratin AE1/AE3 on epithelial tumoral cells (×200).

**Figure 9:**
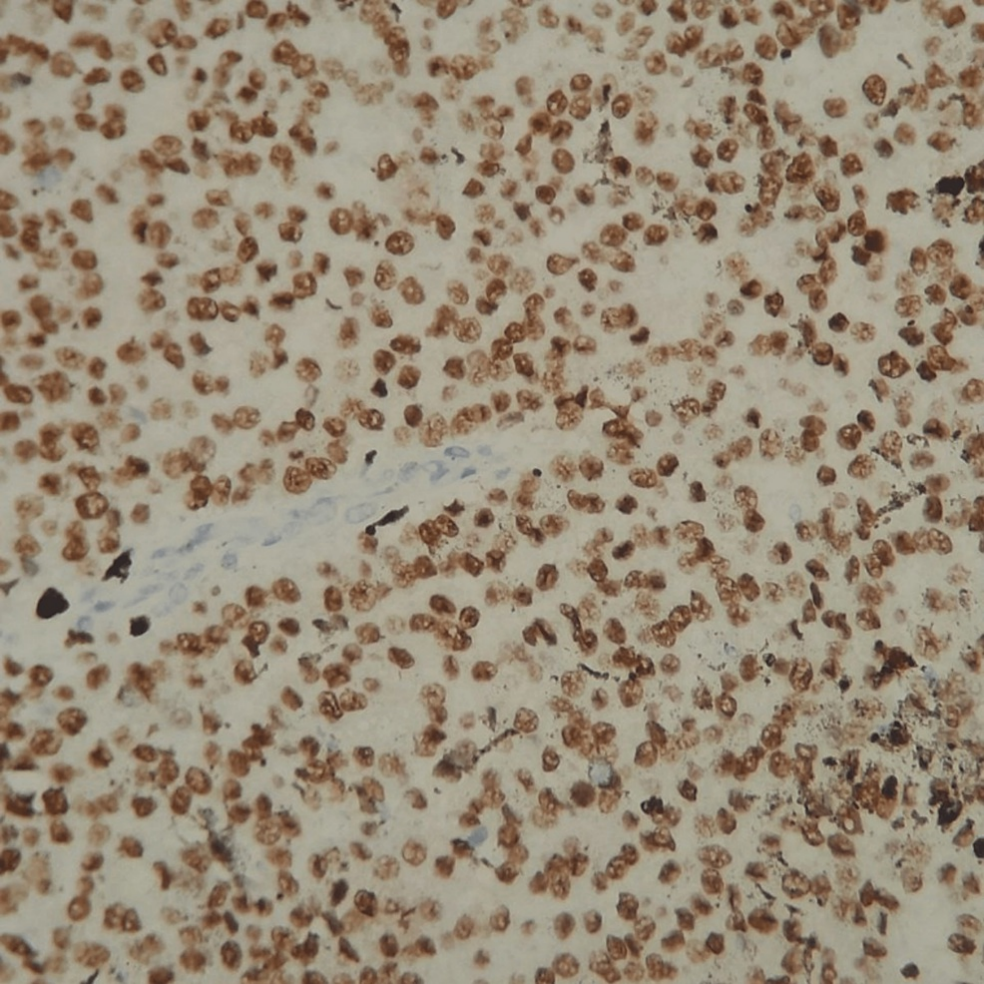
Nuclear positive immunostaining by p63 on epithelial tumoral cells (×200).

**Figure 10:**
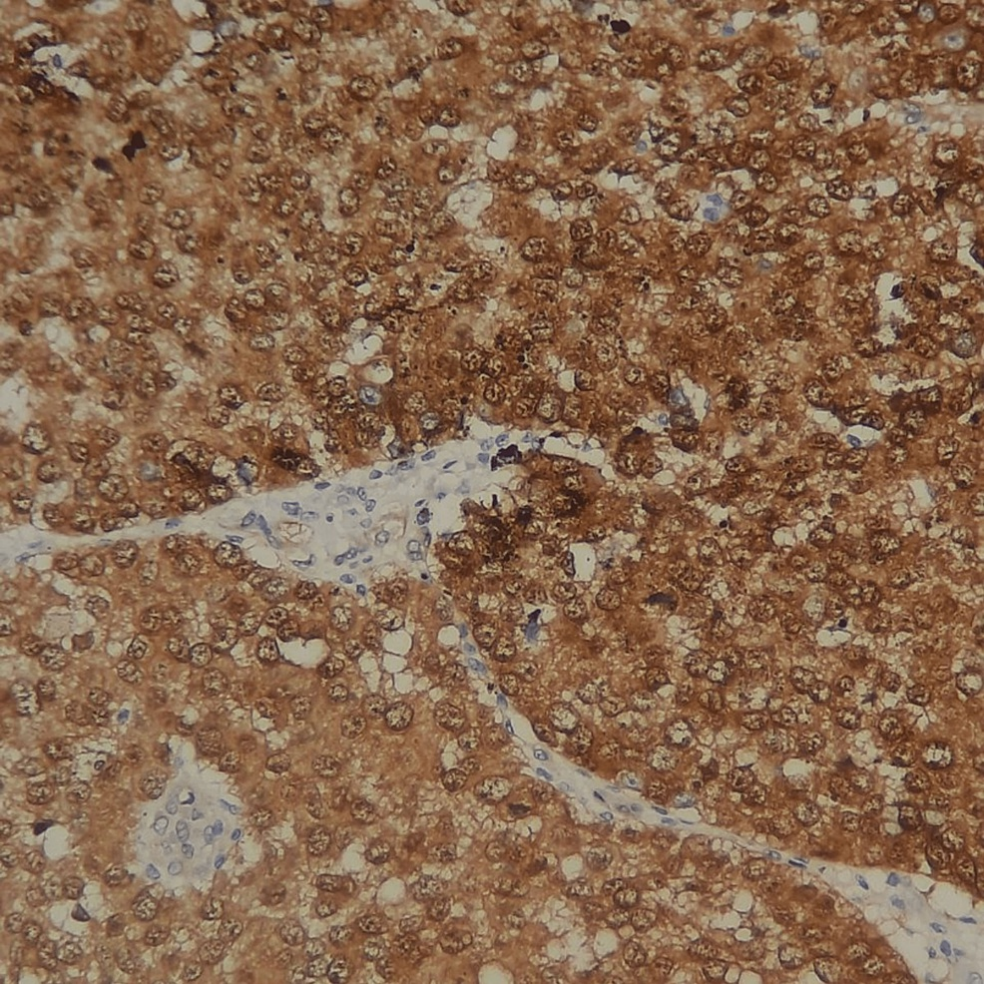
Strong nuclear and cytoplasmic positive immunostaining by beta-catenin on epithelial tumoral cells (×200).

**Figure 11:**
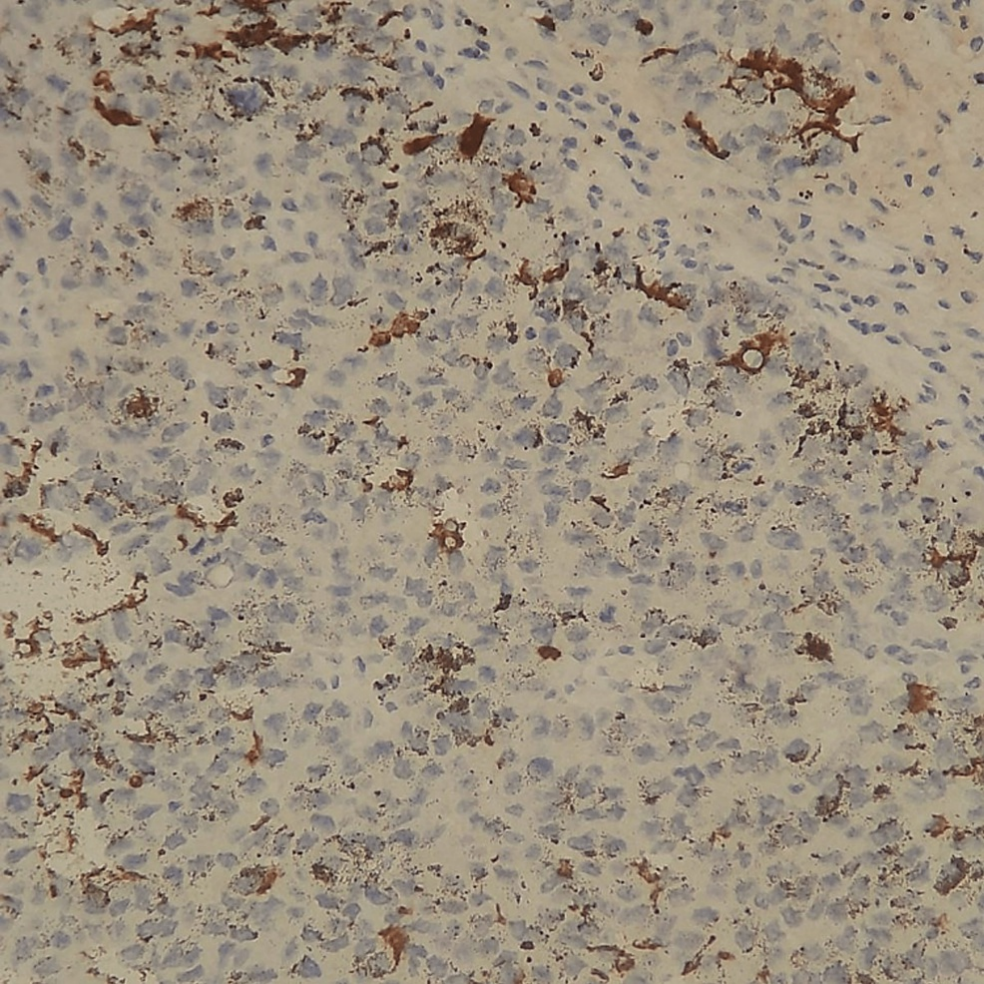
Melanin markers (Melan A and HMB45) highlight non-atypical dendritic melanocytes (×200).

Epithelial tumor cells were basaloid, with a pleomorphic nucleus, prominent nucleolus, and basophilic-reduced cytoplasm. Numerous mitotic figures were noted with atypical ones (22 mitoses/2 mm²) (Figure 5). These cells were mixed with numerous heavily pigmented dendritic melanocytes (Figure 6). Clusters of ghost cells (Figure 7) were noted along with foci of necrosis. On immunohistochemical analysis, tumor cells expressed cytokeratin AE1/AE3 (Figure 8), p63 (Figure 9), and beta-catenin (nuclear and cytoplasmic staining) (Figure 10), while melanin markers (Melan A and HMB45) highlight non-atypical dendritic melanocytes (Figure 11).

All of these findings confirmed the diagnosis of MMM. A cervico-thoraco-abdominal CT scan was indicated to search for lymph node involvement or distant metastasis, which revealed no abnormalities. Carcinological re-excision of the tumoral site for margins was performed, and it is free of tumor residue. No treatment with chemotherapy or radiotherapy was indicated. Follow-up at six months showed no evidence of recurrence or metastasis.

## Discussion

To our knowledge, to date, only 11 cases have been reported in the literature, according to our research using PubMed/Medline, Scopus, and Web of Science databases, with the search terms “malignant melanocytic matricoma” and “matrical carcinoma” [1,3-10]. The epidemiological, clinical, histopathological, immunohistochemical, and behavioral features of the reported cases of MMM are detailed in Table 1.

**Table 1:** Epidemiological, clinical, histopathologic, and immunohistochemical features of reported cases of malignant melanocytic matricoma. BCC = basal cell carcinoma; HPF = high-power field; IHC = immunohistochemistry; SCC = squamous cell carcinoma; NA = not available; NED = not evidence of disease

	Reference (year)	Age (year)/Gender	Location	Size (mm)	Architecture	Connection to the epidermis	Shadow cells	Mitosis	Atypical mitosis	Ulceration	Necrosis	IHC of epithelial cells	IHC of dendritic melanocytes	Treatment	Follow-up	Recurrence
1	Monteagudo et al. (2003)[1]	48/Male	Neck	10	Ill-defined infiltrative nodules and nests	No	Focal	4/10 HFP	NA	No	No	Cytokeratin AE1-AE3, CAM5.2	S-100 protein, HMB45	Surgery	24 months	Yes
2	Monteagudo et al. (2003)[1]	77/Male	Chest	NA	Ill-defined infiltrative nodules and nests	No	Multiple foci	34/10 HPF	NA	Yes	Yes	Cytokeratin AE1-AE3, CAM5.2	S-100 protein, HMB45	Surgery	NA	NA
3	Jani et al. (2008) [3]	77/Male	Bridge of the nose	NA	Infiltrative sheets of cohesive basaloid tumor cells and squamous eddies	No	Focal	Numerous	Yes	No	Yes	BerEP4, P63, +/-cytokeratin 5/6	HMB45, Mrt-1, S-100 protein	Surgery	2 months	NED
4	Limarporn et al. (2012) [4]	81/Male	NA	NA	Nodular growth	No	NA	>20/10 HPF	No	Yes	No	Cytokeratin	S-100 protein, Melan A	Surgery	NA	NA
5	Ardakani et al. (2016) [5]	72/Female	Right elbow	25	Ill-defined with solid and cystic areas and pushing borders	No	Present	>30/10 HPF	No	No	Yes	Diffuse beta-catenin, P63, EMA (focal weak), patchy CK5/6, negative BerEP4	Melan-A, MITF	Surgery	6 months	NED
6	Ardakani et al. (2016)[5]	78/Male	Face	5	Multi-nodular with endophytic growth, pushing borders	No	Present	>50/10 HPF	No	No	Yes	diffuse beta-catenin, p63, focal weak EMA, negative BerEP4	SOX10, Melan-A, MITF	Surgery	6 months	NED
7	Villada et al. (2016) [6]	79/female	Right leg	22	Irregularly shaped dermal nodules, extending into the subcutaneous tissue	No	Present	>10 /10 HPF	Yes	Yes	Yes	Diffuse beta-catenin, negative BerEP4	S-100 protein, Melan A, HMB45	Surgery	NA	NA
8	Ji et al. (2017) [7]	80/Male	Left periauricular	7	Irregular nests and confluent nodules	No	Small foci	>10/10 HPF	Yes	Yes	Yes	AE1/AE3, B-catenin, EMA, p63	SOX10, Melan-A	Surgery	12 months	NED
9	Nielson et al. (2018)[8]	81/Male	Left forearm	NA	Ill-defined, infiltrative	No	Small foci	5/10 HPF	No	No	Yes	Cytokeratin AE1/AE3, cytokeratin 5/6	Melan-A, S-100 protein, HMB45	Surgery	24 months	yes
10	Lehmer et al. (2019) [9]	85/Male	Right cheek	10	Large basaloid nodules	No	Small foci	10/1 HPF	Yes	No	No	Diffuse strong beta-catenin, cytokeratin AE1/AE3, and Ber-EP4 (heterogeneous)	Melan-A	Surgery	NA	NA
11	Melson et al. (2022) [10]	92/Female	Right nasal sidewall	12	Well-circumscribed	No	Present	88/10 HPF	Yes	Yes	No	Diffuse strong beta-catenin	Melan A, SOX10	Surgery	14 months	NED
12	Current case (2023)	86/Female	Right cheek	40	Infiltrative, pigmented tumor arranged in lobules and sheets	No	Focal	22/10 HPF	Yes	Yes	Yes	Cytokeratin AE1/AE3, p63, strong and diffuse beta-catenin	Melan-A, HMB45	Surgery	6 months	NED

The term MMM was first proposed in 2003 by Monteagudo et al. in their study which reported two cases of matrix carcinoma with prominent melanocytic hyperplasia and considered it to be the malignant counterpart of melanocytic matricoma [1]. The latter is a rare benign adnexal tumor first described in 1992 by Carlson et al. [2]. Since then, approximately 20 cases of melanotic matricoma have been reported [2].

On pathogenic findings, the bulb of hair follicles in the anagen phase is known to contain matrix and supramatrix cells, as well as pigmented dendritic melanocytes. Because melanocytes are most important at the beginning of the anagen phase, it is suggested that melanotic matricoma results from hair follicles at an early stage of the anagen phase as a biphasic proliferation of matrix epithelial cells admixed with increased numbers of pigmented dendritic melanocytes and rare foci of ghost cells [10,11]. Therefore, when these features are associated with histological malignancies, such as severe cytological atypia, atypical mitosis, infiltrating growth pattern, necrosis, ulceration, and recurrence [8], the diagnosis of MMM should be considered.

Based on the clinical appearance of this lesion, as it is described since 2003, which appears as a pigmented nodule, occurring on sun-damaged skin, preferentially in the head and neck region (7/11 cases), in the elderly (mean age: 78 years, range: 48-92 years) with a size ranging from 5 mm to 40 mm, the clinical diagnosis is often missed suggesting melanoma, pigmented basal cell carcinoma, seborrheic keratosis, or squamous cell carcinoma (see Table 1) [1,3-10].

On histological examination, MMM must be differentiated from pigmented basal cell carcinoma, trichoblastoma, basal cell carcinoma with matrical differentiation, melanoma, and benign melanocytic matricoma. The severe cytological atypia of MMM argues against trichoblastoma and basal cell carcinoma. Strong diffuse nuclear and cytoplasmic expression of beta-catenin and melanic makers negativity differentiate MMM from melanoma and support matrical differentiation [11].

The treatment of MMM is not yet codified given the rarity of this entity and the absence of a large series investigation. However, the review of the 11 reported cases allowed us to deduce that surgical excision with a free margin is the most reliable option to avoid recurrences. Of the eight cases for which follow-up was available (see Table 1), two presented a recurrence at the resection site, and no lymph node or distant metastasis was revealed in these cases. This lets us reflect on the real aggressiveness of this tumor.

## Conclusions

MMM is an extremely rare malignant skin tumor with matrix differentiation. Clinically, this lesion mimics malignant melanoma or pigmented basal cell carcinoma. Histopathologically, it is characterized by biphasic proliferation of atypical matrix epithelial cells admixed with increased numbers of pigmented dendritic melanocytes and rare foci of ghost cells. The immunohistochemical study makes it possible to confirm the matrix origin of the proliferation by the positivity of cytokeratin AE1/AE3 and strong diffuse beta-catenin and the negativity of the melanotic markers which underline the component of non-atypical dendritic melanocytes. However, the histological criteria of malignancy are largely dominated by cytological atypia, atypical mitosis, infiltrative growth pattern, necrosis, ulceration, and recurrence. Hence, pathologists must have this rare entity in mind when faced with a pigmented lesion of the sun-damaged skin in the elderly with a biphasic epithelial and melanocytic component to avoid misdiagnosis.
